# Fatal dengue virus infection in an unvaccinated traveler

**DOI:** 10.1007/s10096-024-05021-4

**Published:** 2024-12-28

**Authors:** Jonathan Steinke, Veronica Di Cristanziano, Jan-Hendrik Naendrup, Dániel Cadar, Martin Gabriel, Roger Grosser, Noëlle Sieg, Lisa Altenrath, Jesko Welters, Alexander Simonis, Rosanne Sprute, Jorge Garcia Borrega, Gertrud Steger, Jonas Schmidt-Chanasit, Alexander Shimabukuro-Vornhagen, Henning Gruell

**Affiliations:** 1 Department I of Internal Medicine, Center for Integrated Oncology Aachen Bonn Cologne Düsseldorf (CIO ABCD), Faculty of Medicine and University Hospital Cologne, University of Cologne, Cologne, Germany; 2 Institute of Virology, Faculty of Medicine and University Hospital Cologne, University of Cologne, Cologne, Germany; 3 The Institute for Research in Operative Medicine, Faculty of Health, Department of Medicine, Witten/Herdecke University, Cologne, Germany; 4 Department of Arbovirology and Entomology, Bernhard-Nocht-Institute for Tropical Medicine (BNITM), Hamburg, Germany; 5 Department of Virology, Bernhard-Nocht-Institute for Tropical Medicine (BNITM), Hamburg, Germany; 6 Wisplinghoff Laboratories, Cologne, Germany; 7 Institute for Molecular Immunology, University of Cologne, Cologne, Germany; 8 Center for Molecular Medicine Cologne (CMMC), University of Cologne, Cologne, Germany; 9 German Center for Infection Research (DZIF), Partner Site Bonn-Cologne, Cologne, Germany; 10 Faculty of Mathematics, Informatics and Natural Sciences, University of Hamburg, Hamburg, Germany

## Abstract

Dengue is an increasing threat to individuals living in or visiting endemic countries. Effective vaccines have become available, but their use in travelers is typically only recommended to individuals with documented prior infection. We present a fatal case of severe dengue in an unvaccinated traveler without known prior dengue virus infection but longitudinal serologic and molecular evidence for secondary infection. In the context of this case and vaccination guidance for travelers, we discuss pre-vaccination screening, potential implications for travelers to regions with ongoing outbreaks, and emphasize the importance of serostatus-independent vaccines.

**Supplementary Information:**

The online version contains supplementary material available at 10.1007/s10096-024-05021-4.

## Introduction

Rising incidence and expanding vector ranges underscore the growing global challenge of dengue [1]. While most dengue virus (DENV) infections are asymptomatic or result in mild disease, severe dengue is associated with substantial mortality [1, 2]. Secondary infection is common in endemic regions and a risk factor for severe disease that can be mediated by enhancement of infection through antibodies formed in response to prior antigen contact [3, 4]. In contrast, most cases of dengue in travelers from non-endemic areas represent primary exposure [5]. Although rare, fatal dengue in travelers has also mostly been reported in primary infection settings [6].

Two live attenuated vaccines reducing virologically confirmed dengue (VCD) and hospitalizations in individuals living in endemic regions have been approved (CYD-TDV, Dengvaxia^®^; TAK-003, Qdenga^®^) [7, 8]. However, CYD-TDV was associated with an increased risk for severe VCD in study participants seronegative at baseline [8]. Thus, CYD-TDV is typically restricted to individuals with test-confirmed prior DENV infection living in endemic areas. Although CYD-TDV is efficacious in seropositive individuals, pre-vaccination screening requirements have been associated with limited implementation and production has been announced to be discontinued. In contrast to CYD-TDV, TAK-003 is authorized for serostatus-independent use. While overall vaccine efficacy was higher in seropositive individuals, TAK-003 showed protection against DENV-1- and DENV-2-associated VCD over a 4.5-year period in baseline-seronegative participants [7]. However, efficacy against DENV-3 could not be shown in dengue-naïve individuals and low case numbers precluded conclusions about DENV-4 [7].

Safety concerns about dengue vaccines focus on the potential for enhanced disease after vaccine-induced seroconversion and subsequent infection. While antibody-dependent enhancement (ADE) provides a theoretical framework, this concept is difficult to determine in clinical trials and whether it contributes to the risk for dengue-naïve CYD-TDV vaccinees has not been demonstrated. Although no clear safety signals were observed in baseline-seronegative individuals becoming infected after TAK-003 vaccination, a potential for enhanced DENV-3- or DENV-4-mediated disease could not definitively be excluded [7]. Partially based on theoretical concerns about ADE and serotype-restricted efficacy, TAK-003 vaccination of travelers from non-endemic countries is therefore typically recommended only after laboratory-confirmed or anamnestic dengue [9–14]. These criteria and difficult reliable serostatus assessment limit traveler vaccination eligibility, encompassing individuals unaware of prior exposure that are at risk of secondary infection.

## Case presentation

**Fig. 1:**
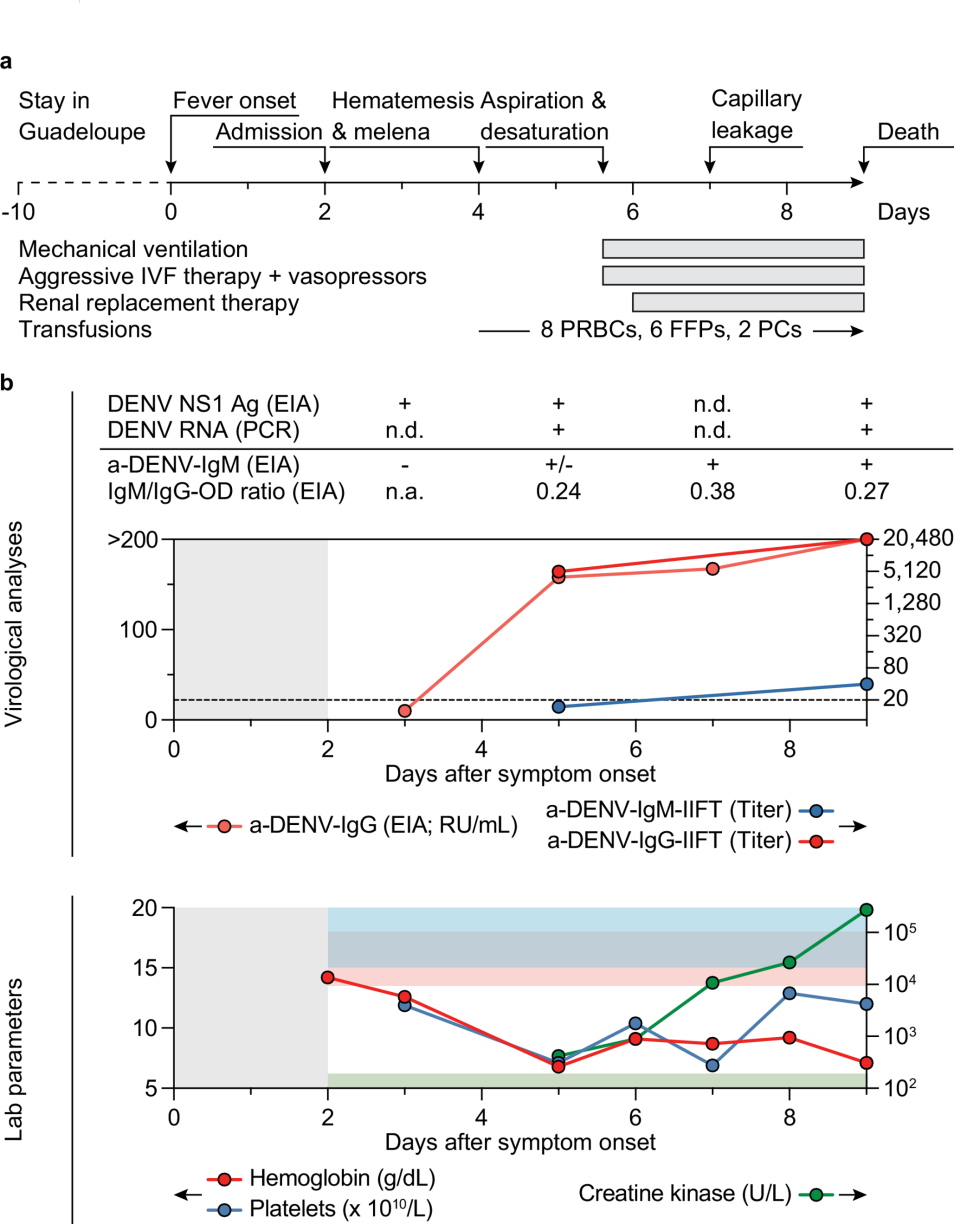
Clinical course and laboratory analyses. **a**, Clinical course and selected medical interventions. **b**, Upper panel indicates results of virological analyses and lower panel indicates selected results of routine laboratory parameters. In the upper panel, the dashed line indicates assay positivity cut-offs (22 RU/mL for a-DENV-IgG and 1:20 for IIFTs). EIAs were performed using ELISAs detecting IgM or IgG antibodies to DENV types 1–4 (Euroimmun Anti-Dengue Virus Type 1–4 ELISA). In the lower panel, shaded areas indicate healthy reference ranges for parameters with colors corresponding to the legend (for platelets and creatine kinase, lower and upper bounds are indicated, respectively). +/-, grayzone; EIA, enzyme immunoassay; FFPs, fresh frozen plasmas; IIFT, indirect immunofluorescence test; IVF, intravenous fluids; n.a., not available; n.d. not determined; OD, optical density; PCs, platelet concentrates; PRBCs, packed red blood cells; RU/mL, relative units per milliliter; U/L, units per liter

Two days after returning home from a 10-day trip to Guadeloupe in early 2024, a 45-year-old previously healthy man from Germany presented with a two-day history of fever, cephalgia, and body aches (Fig. 1a). Physical examination revealed a body temperature of 40.3 °C and lower extremity petechiae. Vital signs were stable (blood pressure 137/78 mmHg, heart rate 100/min, respiratory rate 18/min) and there were no focal neurological signs or cognitive impairment. Laboratory analyses demonstrated thrombocytopenia (119,000 platelets/µL) and leukopenia (2,400 cells/µL). C-reactive protein (1.1 mg/dL), creatine kinase (416 U/L), and serum creatinine (1.5 mg/dL) were slightly elevated. Liver function tests were within normal ranges and hepatomegaly was not palpable. The patient’s travel history included multiple 3-week-trips to Thailand (14, 12, and 6–7 years earlier) and travel to Zanzibar (20 years earlier) without symptoms suggestive of dengue. He was not vaccinated against dengue. Admission was prompted by the overall condition and fever after returning from a tropical region. As expected after travel to Guadeloupe, tests for malaria were negative. However, acute DENV infection was diagnosed by positive NS1 antigen ELISA while IgM and IgG antibodies against DENV 1–4 were negative by ELISA (Fig. 1b). Supportive therapy with acetaminophen and intravenous fluids was initiated.

Two days after admission, sudden melena and hematemesis were associated with severe blood loss (hemoglobin drop from 12.6 to 6.5 g/dL) and rapidly developing hemodynamic instability (Fig. 1b). Two packed red blood cell (PRBC) units and crystalloid fluids were administered and the patient was transferred to a tertiary care hospital intensive care unit with persistent melena and tachypnea (respiratory rate 40/min). Consistent with hemorrhagic shock, laboratory analyses showed lactic acidosis (lactate 15 mmol/L), severe anemia (hemoglobin 6.1 g/dL), thrombocytopenia (89,000 platelets/µL), and coagulopathy (hypofibrinogenemia, PTT prolongation, and INR elevation). Vasopressor therapy was initiated (0.06 µg norepinephrine/kg/min), and platelets and PRBCs were transfused. Gastroscopy showed a gastro-esophageal junction ulcer and hemostatic powder was applied. Blood regurgitation immediately after gastroscopy prompted intubation due to oxygen desaturation. Hypotension and blood loss necessitated transfusion of 3 PRBCs and 4 fresh frozen plasma units as well as intensified fluid and vasopressor therapy (norepinephrine 1.7 µg/kg/min and vasopressin 1.8 U/min). Renal failure (anuria, serum creatinine 5.39 mg/dL) prompted dialysis. Despite control of bleeding, the need for intravenous fluid therapy increased and a fluid balance of > + 6,000 mL/d provided clinical evidence of severe plasma leakage (Fig. 1a). Multiple organ failure was indicated by severe liver damage (bilirubin 5.4 mg/dl, alanine transaminase 14,211 U/L), rhabdomyolysis (creatine kinase 272,940 U/L), and developing left ventricular dysfunction suggestive of cardiomyopathy that was treated with dobutamine (Fig. 1b). Extracorporeal membrane oxygenation was not performed in the context of multiorgan failure. Further hemodynamic deterioration developed in a setting of cardiogenic and capillary leak-related shock, and the patient died nine days after symptom onset.

Blood samples collected five and nine days after symptom onset were positive for DENV RNA by qRT-PCR and DENV NS1 antigen by ELISA. Consistent with epidemiological reports from Guadeloupe, metagenomic sequencing and phylogenetic analysis revealed infection with DENV-2II_F.1 (Supplementary Fig. 1) [15, 16]. While no DENV-reactive antibodies were detected on day 3, increasing and high levels of DENV-reactive IgG could be identified on days 5, 7, and 9 by ELISA (158 RU/mL, 167 RU/mL, and > 200 RU/mL, respectively), and on days 5 and 9 by indirect immunofluorescence (titers of 1:5,120 and 1:20,480, respectively) (Fig. 1b). In contrast, anti-DENV IgM titers remained low both by ELISA (sample/calibrator extinction ratios of 1.0, 1.8, and 1.6 on days 5, 7, and 9, respectively) and indirect immunofluorescence assay (titers of < 1:20 and 1:40 on days 5 and 9, respectively) (Fig. 1b).

## Discussion

While endemic regions bear most of the significant burden of dengue, it is also the most frequent arboviral disease in returning travelers [17]. Severe disease is a rare complication but warrants awareness as increasing incidence and global mobility will likely result in an increase in cases in areas with limited experience in diagnosis and treatment. While effective vaccines have the potential to significantly reduce the risk of severe dengue, our report indicates some of the dilemmas associated with current vaccines and guidance for travelers.

Most recommendations indicate that vaccination can be considered in individuals at relevant risk of exposure who have had dengue in the past [9–14]. However, asymptomatic infection is common among travelers and most individuals may be unaware of prior exposure [18, 19]. Similarly, while our patient did not recall prior dengue-like symptoms despite repeated travel to areas with co-circulation of all DENV serotypes [20], results of virological analyses provided strong evidence for secondary infection [21]. In addition to high IgG titers developing within days and persistently low IgM levels (IgM/IgG ELISA optical density ratios of 0.24 to 0.38), this included persistent DENV-RNAemia in the presence of IgG antibodies. However, despite its potential to reduce secondary DENV infection, dengue vaccination would not generally have been recommended to our patient due to the lack of anamnestic or documented prior exposure [9–14].

Cross-reactivity of antibodies against other flaviviruses (e.g., zika virus) is a known limitation of serological assays for DENV antibodies [22]. While pre-vaccination screening to identify seropositive travelers is often cautioned against due to its potential for false-positive results [9–13], it could aid identification of individuals with unknown prior infection. However, despite clear longitudinal signs for prior exposure, initial testing in our patient did not find DENV-reactive antibodies. Although DENV infection is believed to induce long-lasting humoral immunity against the homologous serotype [23], serological assay sensitivity in convalescent individuals is not 100% and lower after monotypic than multitypic DENV infection [24]. Moreover, anti-DENV-IgG titers after primary infection have been reported to correlate with disease severity and decline over years [25, 26]. While lack of initial sample material prevented confirmation of undetectable anti-DENV antibodies in additional assays, we speculate that asymptomatic infection and no re-exposure over ≥ 6 years may have resulted in a negative serological result. Indicating potential limitations of serological assays, this suggests that pre-vaccination screening may not identify a secondary infection risk to prompt dengue vaccination in all individuals.

DENV-2 was known to be the predominant serotype causing a dengue epidemic in Guadeloupe at the time of the patient’s travel and in the preceding months [15]. As TAK-003 vaccination showed protection from severe DENV-2-mediated disease regardless of serostatus [7], one can speculate that vaccination without information on prior exposure but based on the serotype-specific infection risk may have prevented a fatal outcome in this particular scenario [27].

Travel after asymptomatic DENV infection is likely to become an increasingly common scenario. However, reliable identification of prior infection can be difficult to achieve. While epidemiological data- and risk-based individualized decisions to vaccinate irrespective of known prior infection may provide situation-specific benefit for travelers, vaccines with baseline serostatus-independent efficacy and safety would benefit travelers and individuals in dengue-endemic areas alike.

## Electronic Supplementary Material

Below is the link to the electronic supplementary material.

Supplementary Material 1

## Data Availability

No datasets were generated or analysed during the current study.
