# Supplementary material for: Fatal dengue virus infection in an unvaccinated traveler
Source: Eur J Clin Microbiol Infect Dis. 2024 Dec 28;44(3):747–52. doi: 10.1007/s10096-024-05021-4 (PMC11880070; doi:10.1007/s10096-024-05021-4)
Supplement: Supplementary file 1 — Supplementary Material 1 [file 10096_2024_5021_MOESM1_ESM.pdf]

Supplementary Figure 1

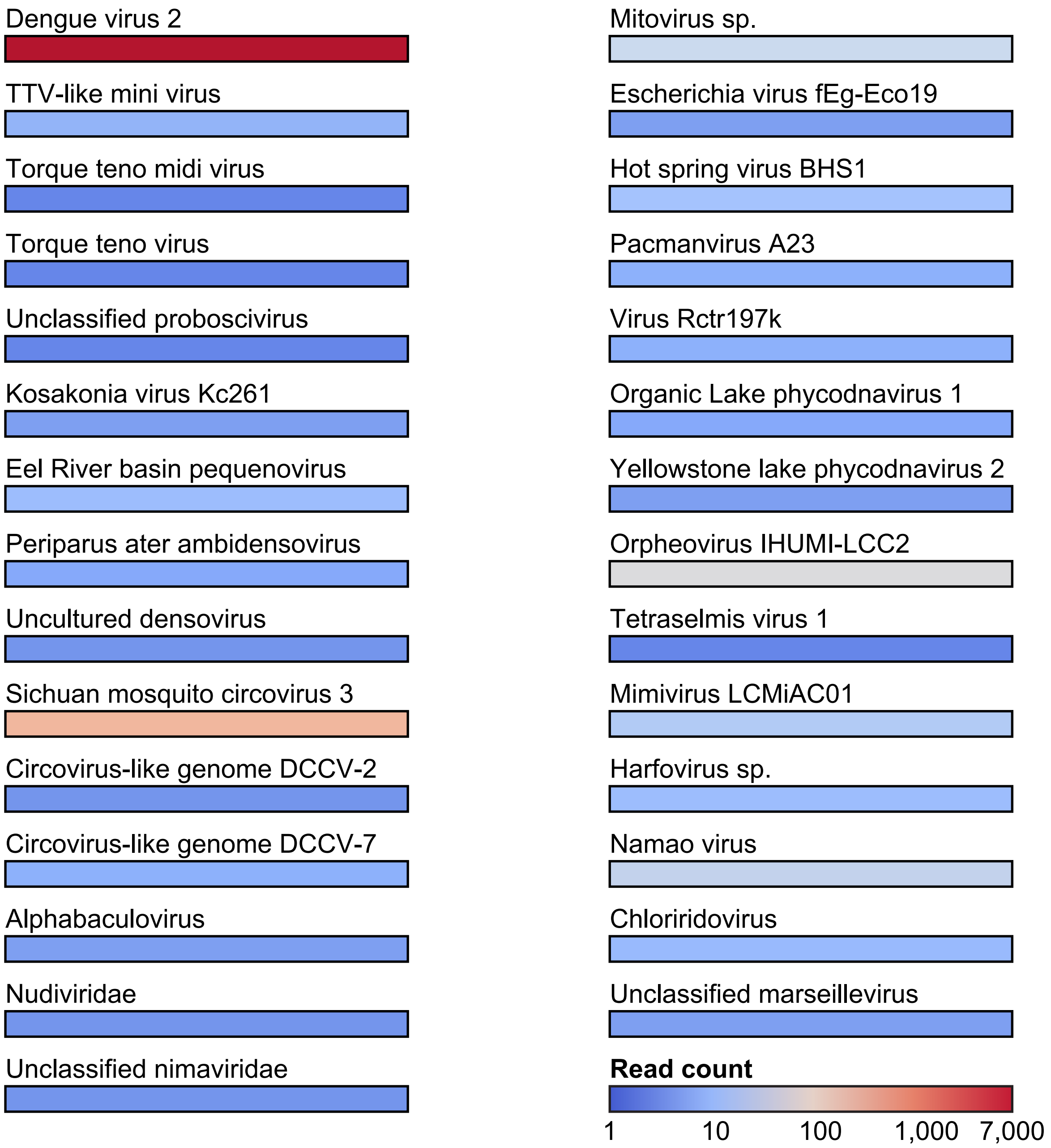

Figure S1. Serum virome composition.
Viral metagenomic sequencing of patient serum sample performed on the NextSeq 2000 platform as described by Cadar *et al.* (doi: 10.1080/22221751.2021.1902752). Colors indicate individual read counts.
